# COVID‐19 vaccination in patients with rheumatic diseases: Vaccination rates, patient perspectives, and side effects

**DOI:** 10.1002/iid3.589

**Published:** 2022-01-31

**Authors:** Yan Kiu Li, Michael Pak Kiu Lui, Lip Long Yam, Chi Shing Cheng, Terence Hon Ting Tsang, Wing Sum Kwok, Ho Yin Chung

**Affiliations:** ^1^ Division of Rheumatology and Clinical Immunology, Department of Medicine The University of Hong Kong Pok Fu Lam Hong Kong; ^2^ Chiron Medical Central Hong Kong

**Keywords:** COVID‐19, immunosuppressants, rheumatic diseases, side effect, vaccination

## Abstract

**Introduction:**

To investigate the vaccination rate, reported side effects, and patient concerns for COVID‐19 vaccination in patients with rheumatic diseases.

**Methods:**

A multicentre cross‐sectional study from rheumatology clinics in two major hospitals in Hong Kong was conducted between June 3, 2021 and October 8, 2021. Patient interviews for demographics, clinical characteristics, vaccination status, reported side effects, and factors influencing decisions about vaccination were supplemented with structured questionnaires.

**Results:**

Out of 1367 patients, 413 (30.2%) had received COVID‐19 vaccination. Side effects were reported in 335 (81.1%) of patients, of which the most common were injection site pain or swelling (66.3%), fatigue (57.1%), fever (19.9%), and headache (19.6%). Multivariate logistic regression models showed that males (odds ratio [OR] = 1.80; *p* < .001), higher education level (OR = 1.64; *p* < .001) and healthcare professionals (OR = 4.5; *p* < .001) were significantly more likely to have received the vaccine. In contrast, patients with hypertension (OR = 0.73; *p* = .04), systemic lupus erythematous (OR = 0.53; *p* < .001), stroke (OR = 0.29; *p* = .01), steroid therapy (OR = 0.59; *p* = .01), and leflunomide therapy (OR = 0.45; *p* = .05) were significantly less likely to be vaccinated. Younger age (age, OR = 0.96; *p* = .003) and messenger RNA (mRNA) vaccines (OR = 4.79; *p* < .001) were associated with more side effects. There was no difference in risk of side effects between specific rheumatic diseases or drug therapies.

**Conclusion:**

COVID‐19 vaccination is associated with no increased risk of side effects in any particular disease or drug therapy, therefore vaccination should be encouraged in patients with rheumatic disease. In addition, younger age is associated minimally, while mRNA vaccine is associated with increased side effects.

## Graphical abstract

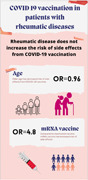

## 1 INTRODUCTION

Severe acute respiratory syndrome coronavirus 2 (SARS‐CoV‐2) as of March 2021 has infected more than 54 million people^1^ and caused over 1.6 million deaths worldwide,^2^ resulting in detriments to global health and economy. The COVID‐19 pandemic has compelled many countries to implement policies for social distancing, face masks, and personal hygiene with the goal of reducing the transmission chain. COVID‐19 vaccination is efficacious in reducing infectivity, and decreasing morbidity and mortality. However, vaccine hesitancy^3^ is all too common, presumably due to worries related to side effects. It is necessary to address these concerns to optimize vaccination rates and achieve herd immunity, especially in patients with rheumatic disease who may be more concerned about side effects risks due to immune dysfunction.

Rheumatic diseases are a group of immune‐mediated inflammatory diseases mainly affecting joints and connective tissues. An English study has found higher mortality due to COVID‐19 in patients with rheumatoid arthritis (RA), lupus, and psoriasis, compared to those without the rheumatic disease (adjusted odds ratio [aOR] of 1.30 [95% confidence interval [CI]: 1.21–1.38]).^4^ Among patients with rheumatic disease, Hyrich et al.^2^ found higher rates of hospitalization due to COVID‐19 in patients on high dose glucocorticoid therapy (≥10 mg/day), with an OR of 2.09, (95% CI: 1.06–3.96). COVID‐19 with the comorbid rheumatic disease may be more severe or even fatal, therefore vaccination is recommended for patients with stable disease status without recent flare‐ups. However, vaccine hesitancy is not uncommon in this group. In a cohort of 1266 patients with rheumatic disease, Rubbert‐Roth et al.^5^ found that 32.2% were uncertain and 13.6% were unwilling to receive COVID‐19 vaccination. Less optimistic findings were suggested by Gaur et al.^6^ that only 54% agreed for vaccination, with major concerns being fear of possible side effects and flare‐ups precipitated by the vaccine.

As there is a lack of data on patient concerns and vaccination side effects in different rheumatic diseases and immunosuppressant therapies, this study explored rates of COVID‐19 vaccination, reported side effects, patient concerns, and strategies for optimization of vaccination in patients with rheumatic disease in Hong Kong.

## 2 METHODS

### 2.1 Study design

This was a prospective cross‐sectional study primarily consisting of interview‐based surveys conducted between June 3, 2021 and October 8, 2021, after the implementation of the COVID‐19 vaccination program in Hong Kong in February 2021. Consecutive patients attending follow‐up at rheumatology clinics in two major public hospitals (Queen Mary Hospital and Grantham Hospital) in Hong Kong were recruited. All participants gave informed verbal consent to the survey study.

### 2.2 Interview‐based survey questionnaire

The questionnaire was developed based on literature reviews and expert discussion including rheumatologists. Interviews based on the questionnaire were conducted in English or Chinese based on the preferences of the participants. Medical doctors or medical students conducted face‐to‐face structured interviews based on the questionnaire. Participants were reassured regarding the confidentiality of their responses to minimize potential bias due to self‐reporting information.

The questionnaire was designed to include three major aspects^1^: participant's characteristics including gender, age, educational level, occupation, smoking history, drinking history, other comorbidities, family history of rheumatic diseases;^2^ details of underlying rheumatic disease(s) including the type of rheumatic disease(s): systemic lupus erythematous (SLE), RA, ankylosing spondylitis (SpA), vasculitis, scleroderma and others such as Sjogren's syndrome, as well as rheumatologic medication(s) before disease flare, change in medication dosage and regimen before/after vaccination, previous disease flare‐up, features of flare‐up; and^3^ COVID‐19 vaccination history including vaccination status, date of vaccination, type of vaccine received (Comirnaty vs. CoronaVac), history of COVID, reasons for getting/not getting vaccinated and reported side effects. For part,^2^ diagnoses and medications were cross‐checked with the clinical management system, a centralized system of electronic medical records of all public hospitals in Hong Kong.

### 2.3 Statistical analysis

Continuous variables were presented as mean ± standard deviation. Categorical variables were presented as frequencies (%). The vaccine uptake rate among the study population and reasons for receiving or not receiving the vaccine were summarized in tables and descriptive graphs, with *χ* ^2^ and *t*‐test used to compare between those two populations, for categorical and continuous variables, respectively. Descriptive analysis regarding vaccine acceptance as well as associations between possible factors and side effects among vaccinated patients were also performed. Multivariate logistic regression analyses were conducted to investigate potential determinants regarding COVID‐19 vaccine acceptance among the study population. Demographic and clinical characteristics of the patients with *p* < .1 from the *χ* ^2^ and *t*‐tests were used as covariates. Similarly, logistic regression models were also built to investigate the potential risk factors for COVID‐19 vaccine side effects. Covariates with a *p*‐value of less than .1 in univariate analysis were added into a “enter” logistic regression model. Results were reported as OR. Multicollinearity was checked through the variance inflation factor scores. *p* Values of less than .05 were identified as statistically significant. R Studio version 4.1.0 was used for all the statistical analyses. List‐wise deletion was applied for missing data.

### 2.4 Ethics approval

The study was approved by the Institutional Review Board of the University of Hong Kong/Hospital Authority Hong Kong West Cluster (reference number UW 21‐279). It was conducted in accordance with the Declaration of Helsinki and the guidance of Good Clinical Practice, November 30, 2006. As our study imposed minimal risks to subjects, the ethics committee waived the need for written informed consent.

## 3 RESULTS

A total of 1307 patients with the rheumatic disease were interviewed. This cohort was characterized by middle age, female predominance, and a high percentage (38.7%) with tertiary education (Table 1). The most common diagnoses were RA (28.6%) and SLE (28.1%), and the most common medications were hydroxychloroquine (30.5%) and prednisolone (29.3%). The most common comorbidities were hypertension (29.5%) and hyperlipidemia (24.5%). None had SARS‐CoV‐2 infection.

**Table 1:** Baseline demographic and clinical characteristics of the study population

	Total (*n* = 1367)	Vaccinated group (*n* = 413)	Nonvaccinated group (*n* = 954)	*p* Value
Age (years)	53.8 ± 14.9	52.9 ± 13.1	54.2 ± 15.5	.053
Male sex (*n*, %)	305/1367 (22.3%)	128/413 (31.0%)	177/954 (18.6%)	<.001
Smoker (*n*, %)	193/1367 (14.1%)	64/413 (15.5%)	129/954 (13.5%)	.34
Drinker (*n*, %)	116/1367 (8.5%)	35/413 (8.5%)	81/954 (8.5%)	.99
Family history of rheumatic diseases	290/1361 (21.3%)	88/412 (21.4%)	202/949 (21.3%)	.98
Higher education level (tertiary education)	526/1358 (38.7%)	205/412 (49.8%)	321/946 (33.9%)	<.001
Healthcare professional (*n*, %)	52/1362 (3.8%)	35/412 (8.5%)	17/950 (1.8%)	<.001
Student (*n*, %)	29/1362 (2.1%)	8/412 (1.9%)	21/950 (2.2%)	.75
Retired/unemployed (*n*, %)	491/1362 (36.0%)	117/412 (28.4%)	374/950 (39.4%)	<.001
Hypertension	403/1367 (29.5%)	93/413 (22.5%)	310/954 (32.5%)	<.001
Diabetes mellitus	127/1367 (9.3%)	32/413 (7.7%)	95/954 (10.0%)	.20
Hyperlipidemia	335/1367 (24.5%)	102/413 (24.7%)	233/954 (24.4%)	.91
Ischemic heart disease	106/1367 (7.8%)	24/413 (5.8%)	85/954 (8.6%)	.08
Stroke	71/1367 (5.2%)	6/413 (1.5%)	65/954 (6.8%)	<.001
History of malignancy	82/1367 (6.0%)	21/413 (5.1%)	61/954 (6.4%)	.35
Type of rheumatic disease a (*n*, %)				
SLE	384/1367 (28.1%)	70/413 (16.9%)	314/954 (32.9%)	<.001
RA	391/1367 (28.6%)	113/413 (27.4%)	278/954 (29.1%)	.50
SpA	267/1367 (19.5%)	116/413 (28.1%)	151/954 (15.8%)	<.001
Vasculitis	45/1367 (3.3%)	16/413 (3.9%)	29/954 (3.0%)	.43
Systemic sclerosis	38/1367 (2.8%)	10/413 (2.4%)	28/954 (2.9%)	.60
Myositis	39/1367 (2.9%)	12/413 (2.9%)	27/954 (2.8%)	0.94
IgG4 disease	15/1367 (1.1%)	3/413 (0.7%)	12/954 (1.2%)	0.39
Other rheumatic diseases	251/1367 (18.4%)	85/413 (20.6%)	166/954 (17.4%)	0.16
Previous history of rheumatic disease flare‐up	807/1365 (59.1%)	256/412 (62.1%)	551/953 (57.8%)	.14
Medications (*n*, %)				
Steroid	401/1367 (29.3%)	76/413 (18.4%)	325/954 (34.1%)	<.001
MTX	286/1367 (20.9%)	93/413 (22.5%)	193/954 (20.2%)	.34
Sulfasalazine	141/1367 (10.3%)	37/413 (9.0%)	104/954 (10.9%)	.28
Leflunomide	54/1367 (4.0%)	10/413 (2.4%)	44/954 (4.6%)	.06
HCQ	417/1367 (30.5%)	109/413 (26.4%)	308/954 (32.3%)	.03
Anti‐TNF	59/1367 (4.3%)	27/413 (6.5%)	32/954 (3.4%)	.01
Azathioprine	80/1367 (5.9%)	22/413 (5.3%)	58/954 (6.1%)	.59
MMF	167/1367 (12.4%)	31/413 (7.5%)	138/954 (14.5%)	<.001

Abbreviations: HCQ, hydroxychloroquine; IgG4, immunoglobulin G4; MMF, mycophenoate mofetil; MTX, methotrexate; *N*, number; RA, rheumatoid arthritis; SLE, systemic lupus erythematous; SpA, spondyloarthritis; TNF, tumor necrosis factor.

^a^ Rheumatic diseases could overlapped.

COVID‐19 vaccination was received by 413 (30.2%) participants. Among them, 263/411 (64.0%) received the messenger RNA (mRNA) (Pfizer‐BioNTech Comirnaty) vaccine and 148/411 (36.0%) received CoronaVac vaccine. The type of vaccine was missed in two patients. The vaccination rate in this study was lowered than that of Hong Kong (60.6% on October 8, 2021).^7^ The vaccinated group was younger, male predominant, with higher education, with more healthcare professionals, with fewer comorbidity, and on less medications. Patients with SpA had higher vaccination rates than SLE (Table 2). The top three reasons for getting vaccinated were protecting self from COVID‐19, protecting others from COVID‐19, and helping to end the pandemic (Table 3). Vaccination was not received in 954 (69.8%) participants, with the top three reasons being fear of side effects, fear of disease flare‐up, and fear of additional side effects due to rheumatic disease (Table 2).

**Table 2:** Reasons for not taking the vaccine

	Total (*n* = 954)
Fear of side effects	705 (74.0%)
Vaccine ineffective	203 (21.3%)
Risk of COVID‐19 is low	298 (31.2%)
Painful injection	61 (6.4%)
Fear of rheumatic disease flare‐up	611 (64.1%)
Decrease vaccine effectiveness due to immunosuppressants	165 (17.3%)
Fear of additional side effects due to rheumatic medications	274 (28.8%)
Increase vaccine risk due to rheumatic diseases	535 (56.1%)
Not enough data on vaccine	388 (40.7%)
Would like to consult a medical professional before vaccine	78 (8.2%)

Abbreviation: *N*, number.

**Table 3:** Reasons for taking the vaccine

	Total (*n* = 413)
Protecting themselves from COVID‐19	375 (90.8%)
Protecting others from COVID‐19	356 (86.2%)
Helping to end the pandemic	305 (73.8%)
Having faith in the vaccine	270 (65.4%)
Advised by a healthcare professional	111 (26.9%)
Government promotion	148 (35.8%)
Advised by family member or friend	129 (31.2%)
Peer pressure	31 (7.5%)
Requested by company	42 (3.1%)

Abbreviation: *N*, number.

Three hundred and thirty‐five (81.1%) of vaccinated participants reported side effects (Table 4), of which the most common were injection site pain or swelling, followed by fatigue, fever, and headache. Other side effects were uncommon and no serious side effects leading to hospitalization or death were found.

**Table 4:** Side effects experienced from COVID‐19 vaccine

	Total (*n* = 413)
Injection site pain/swelling	274 (66.3%)
Fatigue	236 (57.1%)
Headache	81 (19.6%)
Upper respiratory tract symptoms	13 (3.1%)
Fever	82 (19.9%)
Allergic reaction	5 (1.2%)
Chest pain/discomfort	5 (1.2%)
Arthralgia	29 (7.0%)
Lymphadenopathy	3 (0.7%)

Abbreviation: *N*, number.

Multivariate analysis among patients who had taken the COVID‐19 vaccine was performed. Factors associated with vaccination were: male, higher educational level, and healthcare professional. Factors associated with no vaccination were: hypertension, stroke, SLE, on steroid, and on leflunomide (Table 5).

**Table 5:** Multivariate analyses on factors associated with taking the vaccine

	OR (95% CI)	*p *Value	OR (95% CI)	*p* Value
Age	0.99 (0.99; 1.00)	.13		
Male sex	1.97 (1.51; 2.57)	<.001	1.80 (1.31; 2.48)	<.001
Higher education level (tertiary education)	1.93 (1.52; 2.44)	<.001	1.64 (1.26; 2.13)	<.001
Healthcare professional	5.10 (2.82; 9.21)	<.001	4.50 (2.40; 8.45)	<.001
Retired/unemployed	0.61 (0.48; 0.79)	<.001	0.79 (0.59; 1.04)	.09
Hypertension	0.60 (0.46; 0.79)	<.001	0.73 (0.55; 0.99)	.04
Ischemic heart disease	0.66 (0.41; 1.05)	.08	0.86 (0.52; 1.42)	.54
Stroke	0.20 (0.08; 0.47)	<.001	0.29 (0.12; 0.70)	.01
SLE	0.42 (0.31; 0.56)	<.001	0.53 (0.36; 0.77)	<.001
SpA	2.08 (1.58; 2.74)	<.001	1.08 (0.75; 1.54)	.69
On steroid	0.44 (0.33; 0.58)	<.001	0.58 (0.40; 0.85)	.01
On leflunomide	0.51 (0.26; 1.03)	.06	0.49 (0.24; 1.00)	.05
On HCQ	0.75 (0.58; 0.97)	.03	1.17 (0.86; 1.60)	.32
On anti‐TNF	2.02 (1.19; 3.41)	.01	1.01 (0.56; 1.80)	.98
On MMF	0.48 (0.32; 0.72)	<.001	0.93 (0.56; 1.52)	.76

Abbreviations: CI, confidence interval; HCQ, hydroxychloroquine; MMF, mycophenoate mofetil; OR, odds ratio; SLE, systemic lupus erythematous; SpA, spondyloarthritis; TNF, tumor necrosis factor.

Multivariate analysis on the incidence of side effects was also performed. Younger age and Comirnaty vaccine were associated with a higher incidence of side effects. Individual rheumatic diseases, comorbidities, and medications were not associated with excess side effects (Table 6).

**Table 6:** Multivariate analyses on factors associated with experiencing side effects of the vaccine

	OR (95% CI)	*p* Value	OR (95% CI)	*p* Value
Age	0.94 (0.92; 0.96)	<.001	0.96 (0.94; 0.99)	.003
Male	0.47 (0.29; 0.78)	.004	0.62 (0.33; 1.17)	.08
Smoker	0.48 (0.26; 0.88)	.02	0.60 (0.28; 1.28)	.19
Drinker	0.55 (0.25; 1.20)	.13		
Pfizer‐BioNTech Comirnaty vaccine	5.99 (3.48; 10.32)	<.001	4.79 (2.68; 8.56)	<.001
SLE	1.99 (0.91; 4.34)	.09	1.60 (0.66; 3.89)	.30
RA	1.11 (0.64; 1.95)	.71		
SpA	0.99 (0.57; 1.72)	.98		
Vasculitis	0.50 (0.17; 1.47)	.21		
Systemic sclerosis	0.93 (0.19; 4.47)	.93		
Myositis	0.45 (0.13; 1.54)	.21		
IgG4 disease	0.11 (0.01; 1.27)	.08	0.15 (0.01; 2.95)	.21
Hypertension	0.69 (0.39; 1.20)	.18		
Diabetes mellitus	1.01 (0.40; 2.54)	.98		
Hyperlipidaemia	0.80 (0.46; 1.39)	.43		
Ischemic heart disease	0.68 (0.26; 1.78)	.43		
Stroke	0.23 (0.45; 1.14)	.07	0.34 (0.05; 2.55)	.30
History of malignancy	0.56 (0.21; 1.50)	.25		
On steroid	0.70 (0.38; 1.27)	.24		
On MTX	1.41 (0.75; 2.65)	.29		
On sulfasalazine	1.00 (0.42; 2.36)	1.00		
On leflunomide	2.13 (0.27; 11.03)	.48		
On HCQ	0.97 (0.55; 1.69)	.91		
On anti‐TNF	1.36 (0.46; 4.06)	.58		
On azathioprine	0.48 (0.19; 1.21)	.12		
On MMF	1.23 (0.46; 3.31)	.68		

Abbreviations: CI, confidence interval; HCQ, hydroxychloroquine; IgG4, immunoglobulin G4; MMF, mycophenoate mofetil; MTX, methotrexate; OR, odds ratio; RA, rheumatoid arthritis; SLE, systemic lupus erythematous; SpA, spondyloarthritis; TNF, tumor necrosis factor.

## 4 DISCUSSION

COVID‐19 vaccination rates are lower in patients with rheumatic disease compared with the general population. Associated factors include SLE, presence of comorbidities, and immunosuppressant therapy. From the patients' perspective, the most common concerns centered on fear of side effects and disease flare‐ups. However, no specific disease or drug therapy was associated with additional side effects.

The vaccination rate in rheumatic patients was 30.2%, lower than in the general population in Hong Kong. Reports on the willingness of vaccinations in rheumatic patients varies. A Dutch study found that COVID‐19 vaccination had similar odds ratios amongst different autoimmune diseases and with the control (nonrheumatic) group.^8^ Similarly, seasonal influenzae vaccination rates were no different between individual rheumatic diseases.^9^ In contrast, studies from India and Turkey^10^, ^11^ found rheumatic patients were less willing to receive the COVID‐19 vaccine, more similar to the results of this study. As COVID‐19 vaccine acceptance in Hong Kong has been consistently low.^12^, ^13^, ^14^ Vaccine hesitancy is one of the most challenging public health issues in our city, yet is the most effective preventive method of halting the transmission chain.^15^ Apart from public health policies and the local prevalence of COVID‐19, this study explored the role of patient perspectives.

Fear of side effects was one of the main reasons for declining COVID‐19 vaccination, not unlike regional findings from an Italian.^16^ The development of COVID‐19 vaccines in 2021 was fast‐tracked especially for the pandemic, and mRNA vaccines were a first. Although vigorous safety testing was done in the general population,^17^ little data exists in disease subgroups.

Common reasons for vaccination hesitancy centered around fears of increased side effects due to rheumatic disease or disease flare‐up, both of which have been reported in previous studies.^8^, ^16^ Although not studied in this report, mRNA vaccine could induce adaptive immune response via Toll‐like receptor 7 and has a theoretical risk of triggering an underlying autoimmune disease.^18^ In this study, reported side effects were the same between individual diseases and immunosuppressant therapies. On the other hand, COVID‐19 in patients with rheumatic disease on glucocorticoid or conventional (c‐) disease‐modifying antirheumatic drug therapy had increased hospitalization and mortality,^15^ highlighting the need for early vaccination in this group of patients. In addition, insufficient long‐term data on the COVID‐19 vaccine as an important reason for vaccine hesitancy in this study was also found in a Dutch study,^8^ which found greater than 60% declined due to this reason. Discussions about COVID‐19 vaccinations supported by local real‐life data can alleviate fears and encourage informed choices.

Another common reason for the low vaccination rates in this study group was the overall low risk of COVID infection in Hong Kong, a region that has adopted a zero‐COVID policy achieved by strict quarantine for travelers in designated hotels, compulsory mask‐wearing in public areas, contact tracing, rapid lockdowns, and mass testing. This policy has allowed the elimination of the effects of COVID in rheumatic patients in this study.^19^

Multivariate regression analysis showed that patients with SLE were less likely to get vaccinated. Compared to inflammatory arthritis, SLE frequently manifests with a more severe and potentially life‐threatening disease, therefore, vaccination hesitancy is not unexpected. Males were more likely to get vaccinated, which was also found in a systemic review published in January 2021.^20^ The contextual factor could be a reason. For example, female patients may wish to defer vaccination due to current or planned pregnancy. Healthcare personnel and higher education level had higher vaccine acceptance. A study in China also reported that high educational attainment at or above college or university level were associated with willingness to be vaccinated.^21^ The most common reasons include fear of COVID‐19, self‐perceived risk of infection, protection of self, family and patients, and intention to contribute to achieving herd immunity.^22^ Possible contributing factors not addressed in this study include socioeconomic status, ethnicity, government policy, local prevalence of COVID‐19, regional hygiene practices. Globally, the proportion of the population with the intention to vaccinate varied greatly, from China (91%), France (76%), to the United States (54%),^20^ suggesting the influence of government policies on vaccination rate.

Safety profiles of the two vaccines available in Hong Kong were compared. The CoronaVac vaccine is a more conventional inactivated vaccine. The Pfizer‐BioNTech Comirnaty is a newer mRNA vaccine in which safety have been demonstrated by multinational placebo‐controlled, observer‐blinded, pivotal efficacy trials.^17^ However, similar data in Asian populations is limited. In this study, more reported side effects were found in younger patients, and in patients who received the Comirnaty vaccine. Having said that, two‐dose regimen of the Comirnaty vaccine conferred 95% protection against COVID‐19,^17^ in contrast to 83% for CoronaVac.^23^ The choice of type of COVID‐19 vaccine should be based on risk‐benefit analysis. All side effects were minor with none requiring hospitalization in our study. Although younger age was found to associate with a higher risk of having side effects, the risk reported in our study was minimal.

International and local guidelines advocate for universal COVID‐19 vaccination in patients with rheumatic disease because benefits outweigh risks. The Asia Pacific League of Associations for Rheumatology COVID‐19 task force recommends vaccination in all rheumatology patients irrespective of immunosuppressive therapy because no evidence has been shown of attenuation of immune response.^24^ The American College of Rheumatology also recommends COVID‐19 vaccination in patients with rheumatic and musculoskeletal disease,^25^ although acknowledging a risk of disease flare or worsening of rheumatic disease.

Findings in this study may guide strategies for optimization of vaccination rates in this group. It is also relevant for COVID‐19 vaccination boosters in the future. Approximately 64% of nonvaccinated patients reported a lack of information as a barrier. Among vaccinated patients, nonhealth reasons for getting vaccinated included ease of future travel, and mandatory requirements in the workplace. Patients' perspectives provide valuable information to guide targeted policymaking that is effective in increasing vaccination rates.

A major limitation is the cross‐sectional design which precluded data of possible delayed side effects of the COVID‐19 vaccine. The absence of a control group also prevented comparisons with other diseases or the community. Patients' self‐reported symptoms and side effects were at risk of recall bias. More research should also be conducted to determine the factors for COVID‐19 vaccination acceptance and long‐term side effects in different rheumatic diseases.

This study was the first to provide a descriptive analysis, exploration of associated factors, and side effects of COVID‐19 vaccination in patients with rheumatic disease. Although local consensus guidelines recommend vaccination in this group,^26^ this study nonetheless has real‐life data that guides clinical management as well as evolving healthcare policies.

## 5 CONCLUSION

COVID‐19 vaccination is associated with no increased side effects between individual rheumatic diseases and immunosuppressant drugs. COVID‐19 vaccination should be encouraged in rheumatic patients as the benefits outweigh potential risks.

## CONFLICT OF INTERESTS

The authors declare that there are no conflict of interests.

## AUTHOR CONTRIBUTIONS

*Study conception and design*: Ho Yin Chung. *Acquisition of data*: Yan Kiu Li, Michael Pak Kiu Lui, Lip Long Yam, Chi Shing Cheng, Terence Hon Ting Tsang, Wing Sum Kwok. *Analysis and interpretation of data*: Yan Kiu Li, Ho Yin Chung. *Drafting the article*: Yan Kiu Li, Michael Pak Kiu Lui, Ho Yin Chung. *Revising the article*: Wing Sum Kwok, Ho Yin Chung.

## Data Availability

Data are available from Dr. Ho Yin Chung upon reasonable request.
